# MicroRNA-210 induces endothelial cell apoptosis by directly targeting PDK1 in the setting of atherosclerosis

**DOI:** 10.1186/s11658-017-0033-5

**Published:** 2017-01-25

**Authors:** Ying Li, Chunyan Yang, Lili Zhang, Ping Yang

**Affiliations:** 1 Department of Cardiology, China-Japan Union Hospital of Jilin University, 130033 Changchun, China; 2 Department of Neonatology, The First Hospital of Jilin University, 130021 Changchun, China; 3 Department of Ultrasonography, Eastern Division of First Hospital of Jilin University, 130021 Changchun, China

**Keywords:** Atherosclerosis, miR-210, PDK1, Endothelial cell apoptosis, ApoE (-/-)

## Abstract

**Background:**

Atherosclerosis is a chronically inflammatory disease and one of the leading causes of deaths worldwide. Endothelial cell apoptosis plays a crucial role in its development. Several microRNAs (miRNAs) are reportedly involved in atherosclerotic plaque formation, including miRNA-210 (miR-210). However, the underlying mechanism of its role in endothelial cell apoptosis during atherosclerosis is still largely unknown.

**Methods:**

A mouse model with atherosclerosis induced by a high-fat diet (HFD) was built in ApoE (-/-) mice. The levels of endothelial cell apoptosis were determined via flow cytometry. The expressions of miR-210 and PDK1 in purified CD31+ endothelial cells from mouse aorta were measured via RT-qPCR and western blot. Binding between miR-210 and the 3′-untranslated region (UTR) of PDK1 mRNA was predicted using bioinformatics analyses and confirmed with a dual luciferase reporter assay. The effects of miR-210 were further analyzed in an in vitro model using human aortic endothelial cells (HAECs) treated with oxidized low-density lipoprotein (ox-LDL).

**Results:**

We found that the HFD mice developed atherosclerosis in 12 weeks and had a significantly higher percentage of endothelial cell apoptosis. The upregulated level of miR-210 in the HFD mice and HAECs inversely correlated with the level of PDK1. Inhibiting miR-210 expression significantly reduced HAEC apoptosis, as evidenced by the results of the MTT and flow cytometry experiments. Further analysis identified PDK1 as the target of miR-210 and showed that PDK1 overexpression reversed the pro-apoptotic effect of miR-210 through mediation of the P13K/Akt/mTOR pathways.

**Conclusion:**

Our study suggests a novel role for miR-210 in the progression of atherosclerosis through the regulation of endothelial apoptosis. This indicates that miR-210 might have potential in treatment of atherosclerosis.

## Background

Atherosclerosis, which is an inflammatory disease and a predominant cause of cardiovascular disorders, is a worldwide issue due to the prevalence of diets high in saturated fats and lipids [1, 2]. In the large arteries, atherogenic lipoproteins, especially low-density lipoprotein (LDL) cholesterol, build up to gradually form atherosclerotic plaques, which have been proven to be involved in atherosclerosis-related morbidity [3, 4].

Apolipoprotein E (ApoE) is a 34-kDa secreted protein that has been shown to have anti-atherosclerosis activity: it targets abundant lipoproteins in arteries and is capable of efficiently removing them [5–7]. Investigations of ApoE (-/-) mice have verified the crucial role of ApoE in protection from atherosclerosis [8]. ApoE (-/-) mice fed with high-fat diets (HFD) display high lipid levels, excessive cholesterol in the blood vessels and atherosclerotic symptoms [9], making this an acknowledged model for investigating atherosclerosis [10].

The endothelium functions as an effective mediator to regulate the vascular system, with roles in processes such as hemostasis, cell cholesterol, hormone trafficking, signal transduction and inflammation [11, 12]. Convincing evidence indicates that dysfunction of the endothelium is associated with various vascular diseases, including diabetes mellitus, arterial thrombosis and hypercholesterolemia [13]. Endothelial cell (EC) apoptosis and death can destroy the structure of plaques, and result in the deposition of local lipids and finally in atherogenesis [14, 15]. Preventing EC apoptosis has garnered considerable attention as a novel means of treating atherosclerosis [16, 17].

MicroRNAs (miRNAs), are small (18–22 nucleotides) noncoding RNAs that regulate gene expression by the binding of the 3′-untranslated regions (3′-UTR) of target genes post-transcriptionally, this suppresses gene expression [18]. Emerging evidence suggests that miRNAs could be effective therapeutic targets for complex human diseases, including tumorgenesis, lymphopoiesis and angiogenesis [19–21].

Preclinical studies have shown that miRNAs also play pivotal roles in the pathogenesis of atherosclerosis [22, 23]. A recent study showed overexpression of miR-210 in the serum samples of patients with arteriosclerosis obliterans [24]. Raitoharju et al. further demonstrated that miR-210 was upregulated in human atherosclerotic plaques and might be involved in the process of atherosclerosis [25]. However, the regulatory mechanism for miR-210 in the setting of atherosclerosis remains unclear.

In this study, we found that miR-210 was upregulated by directly targeting 3-phosphoinositide-dependent protein kinase-1 (PDK1) in vascular endothelial cells in an atherosclerosis mouse model and in human aortic endothelial cells (HAECs) treated with oxidized low-density lipoprotein (ox-LDL). Repression of PDK1 due to miR-210 upregulation was found to critically contribute to endothelial apoptosis in the setting of atherosclerosis. Our findings also showed that PDK1 is an essential miR-210 target in regulating endothelial apoptosis of atherosclerosis by inhibiting P13K/Akt/mTOR signaling activation. This study demonstrates that inhibiting miR-210 expression in endothelial cells may be a promising therapeutic approach for vascular diseases such as atherosclerosis.

## Methods

### Animal models and quantification of atherosclerotic lesions

All experimental procedures were performed according to the guidelines for the Care and Use of Laboratory Animals of Jilin University, Changchun, China. Male 6-week old ApoE (-/-) mice were obtained from the Animal Center of Jilin University and housed under pathogen-free animal room conditions with a 12 h light and dark cycle and a controlled temperature of 25 °C. The animals were randomly divided into two groups of 10, with the control group fed a normal chow diet (NOR group) and the experimental group maintained on a high-fat diet (HFD) for 12 weeks to induce atherosclerosis (HFD group). After 12 weeks, the aortas were carefully excised from the mice. The aortic roots were immediately fixed with 4% paraformaldehyde, embedded in an optimum cutting temperature (OCT) compound, and cut into 7 μm thick cross-sections.

H&E staining was used to examine the atherosclerotic lesions of the aortic root. The number of lesions in each cross-section was counted using the grid on the microscope eyepiece (20 × 20 μm). The length of a lesion along the aortic perimeter and the average thickness were determined and multiplied to obtain a cross-sectional area in μm^2^.

The lipid deposition was stained with an Oil Red O staining kit (Sigma-Aldrich) according to the manufacturer’s instructions and observed through an Olympus reverse microscope. Briefly, the cross-sections of aortic sinuses (7 μm) were fixed with 4% paraformaldehyde, and then stained with Oil Red O. The lipid deposits in the plaques were stained red. The total lesion area was quantified in each group using computer-assisted quantitative analysis (ImageJ).

### RNA isolation and quantitative RT-PCR

Total RNA was isolated from CD31+ endothelial cells. After washing with ice-cold PBS, the aorta was removed and flushed with TRIzol reagent (Invitrogen) using an insulin syringe. The eluate was collected in a 1.5 ml tube and prepared for RNA extraction. Total RNA and miRNA were extracted from tissue or cultured cells with RNeasy and miRNeasy Mini kits (Qiagen), respectively. Complementary DNA (cDNA) was synthesized from the extracted RNA using a High-Capacity cDNA Reverse Transcription kit (Applied Biosystems). RT-qPCR was subsequently performed in triplicate with a SYBR Green PCR Kit (Takara Bio) to quantify the mRNA and miRNA. U6 and GAPDH were used as internal controls to normalize miRNA and mRNA expressions, respectively. The primers for miR-210 detection were designed by Genechem Co., Ltd. The 2^-△△Ct^ method was applied to analyze the relative mRNA expression levels. The sequences of primers were: PDK1 forward: 5′-AGGCAAAGGAAGTCCATCTCA-3′, reverse: 5′-CCCATGCATTTGTGCCTACC-3′; GAPDH forward: 5′-CCCATGTTCGTCATGGGTGT-3′, reverse: 5′-CCCATTCCCCAGCTCTCATA-3′.

### Cell culture and transfection

Normal human aortic endothelial cells (HAECs) were obtained from the American Type Culture Collection (ATCC) and cultured in endothelial cell medium supplemented with endothelial cell growth factors, 5% fetal bovine serum (FBS; Invitrogen) and 1% penicillin/streptomycin (Invitrogen). The cells were maintained at 37 °C with 5% CO_2_. To disturb miRNA expression, HAECs were transiently transfected with miR-210 mimics, miR-210 inhibitors or null controls (Genechem) using Lipofectamine 2000 reagent (Invitrogen) according to the manufacturer’s instructions. To upregulate PDK1 expression, PDK1-overexpressing plasmid was synthesized at Genechem and transfected into HAECs using Lipofectamine 2000. At a point 24 h post-transfection, the cells were treated with or without 50 μg/ml oxidized low-density lipoprotein (ox-LDL, Solarbio Bio-Technolgy) for a further 24 h.

### Cell viability assay

The 3-(4,5-dimethylthiazol-2-yl)-2,5-diphenyltetrazolium bromide (MTT) assay was performed to evaluate the cell viability of HAECs. Cells were seeded in 96-well plates with 3 × 10^4^ cells per well prior to transfection or drug treatment for 24 h. Twenty microlitre of MTT solution (5 mg/ml; Sigma-Aldrich) was added to each well and incubated for 4 h at 37 °C. One-hundred-fifty microlitre of dimethyl sulfoxide (DMSO) was added to dissolve the formazan crystals. Absorbance at 570 nm was measured using a microplate reader (ThermoFisher).

### Western blotting

The cells were harvested and lysed with RIPA buffer containing protease and phosphase inhibitors (Sigma-Aldrich). After centrifugation at 12,000 rpm for 10 min at 4 °C, the supernatants were collected and quantified using the Bradford assay (Bio-Rad). The proteins were then separated using SDS-PAGE and transfected to PVDF membranes (Bio-Rad). After blocking with 5% non-fat milk for 1 h at room temperature, the membranes were incubated with primary antibodies, including anti-PDK1 (ab110025; Abcam), anti-p-Akt (ab38449), anti-Akt (ab32505), anti-p-mTOR (ab109268), anti-Bcl2 (ab692), anti-caspase 3 (ab32351), anti-caspase 9 (ab32539), and anti-GAPDH (ab8245) followed by incubation with the secondary HRP-conjugated antibody (Cell Signaling). The bands were scanned using an enhanced chemiluminescence system and protein intensity was quantified with Image-Pro Plus 6.0 software (Media Cybernetics). GAPDH was used as the internal control.

### Flow cytometry

For isolation of cells bearing the endothelial cell marker CD31+, the aorta was dissociated with 10 μg/ml trypsin (Sigma-Aldrich) and 2.5 mg/ml collagenase II for 35 min. The dissociated single cells were incubated with PE-cy7-CD31 (Becton-Dickinson Biosciences) and then sorted on a FACScan flow cytometer (Becton-Dickinson Biosciences). The CD31+ cells or cultured cells were re-suspended and double stained with FITC-Annexin V and propidium iodide (PI) from a FITC Annexin V Apoptosis Detection Kit I (Becton-Dicknson Biosciences), and cell apoptosis was analyzed using a FACScan flow cytometer equipped with Cell Quest software (Becton-Dickinson Biosciences).

### Luciferase reporter assay

miRNA targets were predicted using algorithms (including PicTar, Target Scan, microRNA). The wild-type and mutant 3′-UTR of PDK1 reporter plasmids were purchased from Genechem. HAECs were seeded in a 24-well plate and co-transfected with 0.5 μg plasmid and miR-210 mimics or miR-210 inhibitors or negative controls using Lipofectamine 2000 reagent. Renilla luciferase was used as an internal control. At a point 48 h post-transfection, the cells were harvested and a Dual-Luciferase Reporter Assay System Gene Assay Kit (Promega) was used to evaluate the relative luciferase activities according to the manufacturer’s instructions.

### Statistical analyses

The data in this study are shown as the means ± standard deviation (SD) and analyzed using GraphPad Prism 5.0 (GraphPad Software). Statistical differences between the different groups were calculated using Student’s *t*-test. The correlation of miR-210 and PDK1 were evaluated by Peason’s correlation coefficient analysis. A value of *p* < 0.05 was considered statistically significant. Each experiment was performed at least 3 times.

## Results

### HFD induces atherosclerosis in ApoE (-/-) mice

**Fig. 1:**
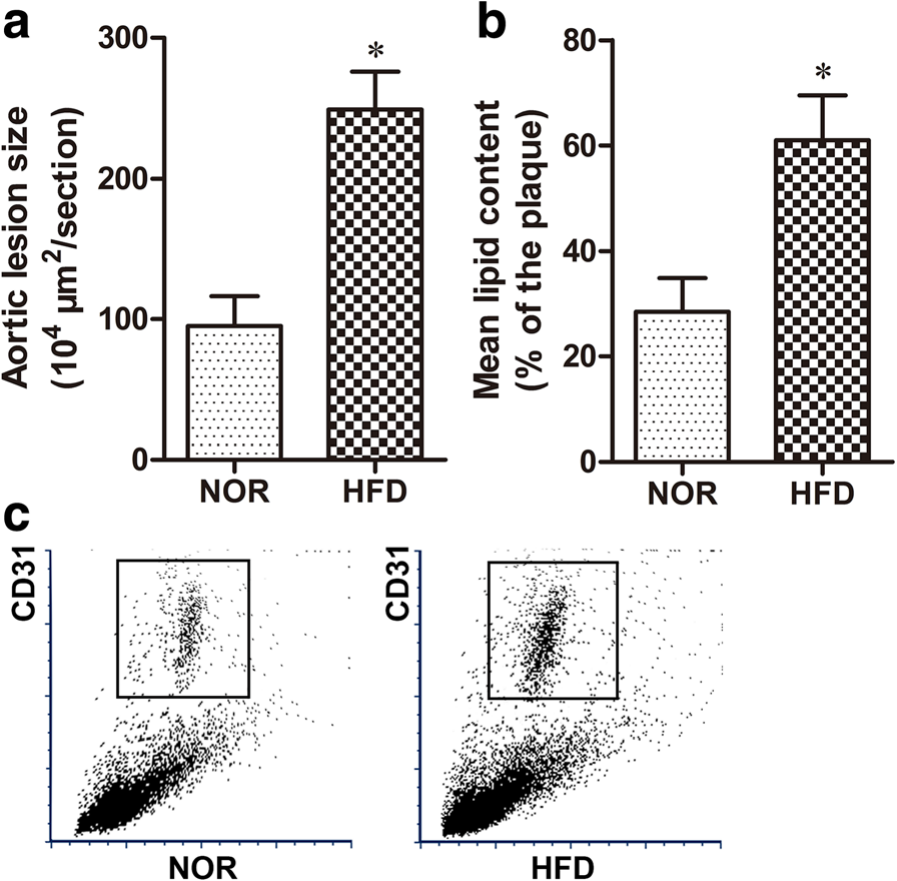
HFD-induced atherosclerosis in ApoE (-/-) mice after 12 weeks. **a** H&E staining and quantification of the plaque area of the aortic sinus. **b** Oil Red O staining and quantification of atherosclerotic lesions in the aortic sinus (lipid deposits stained red). **c** The CD31+ endothelial cells isolated from aortas using flow cytometry. *n* = 10 for each group. *p < 0.05

The HFD group ApoE (-/-) mice spent 12 weeks on a high-fat diet (HFD) while the NOR group ApoE (-/-) mice received a normal diet. As shown in Fig. 1a and b, analysis of H&E- and Oil Red O-stained histological sections of the aortic sinus revealed that atherosclerotic lesions and lipid content were significantly higher in the HFD group (*p* < 0.05) than in the NOR group, suggesting that the HFD successfully induced atherosclerosis in ApoE (-/-) mice. To analyze endothelial cell apoptosis in ApoE (-/-) mice, we dissociated the mouse aorta and purified endothelial cells based on CD31+ labeling via flow cytometry (Fig. 1c).

### HFD induces endothelial cell apoptosis in ApoE (-/-) mice

**Fig. 2:**
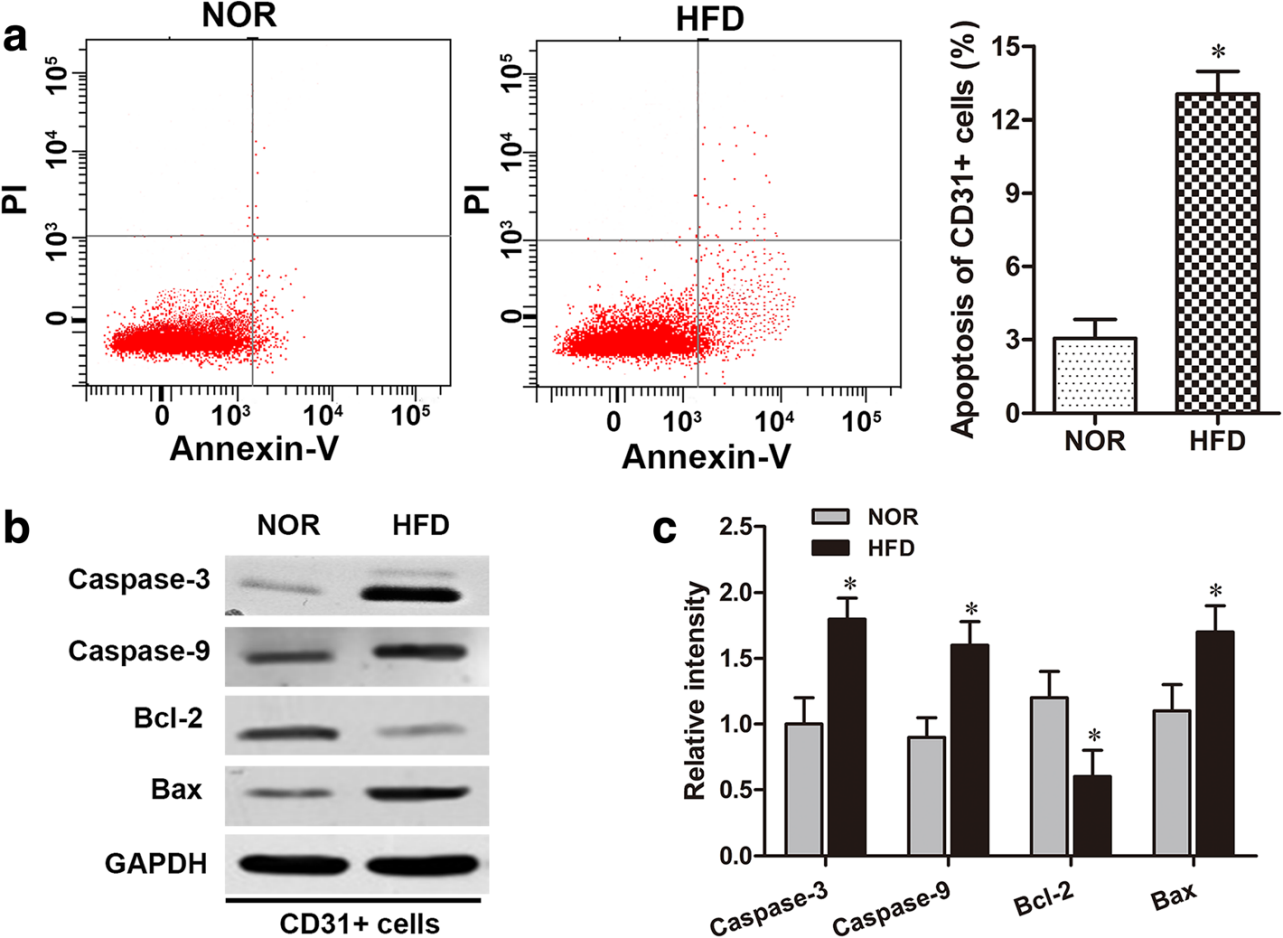
HFD induces endothelial cell apoptosis in ApoE (-/-) mice. **a** The proportions of CD31+ cell apoptosis were measured using the flow cytometry assay in the HFD and NOR groups. **b** The protein levels of Bax, caspase-9, caspase-3 and Bcl-2 in the CD31+ endothelial cells of the HFD and NOR groups were detected via western blot. **c** The protein expressions mentioned in (b) were normalized to GAPDH. *p < 0.05

The apoptosis rate of CD31+ endothelial cells was assessed via flow cytometry. The apoptosis assay revealed that HFD significantly increased the percentage of CD31+ endothelial cell apoptosis in the aorta (Fig. 2a) compared with that in NOR mice (*p* < 0.05). Western blot results for CD31+ endothelial cells also showed that the levels of crucial pro-apoptosis proteins, such as Bax, caspase-9 and caspase-3, had notably increased, while the level of the anti-apoptosis protein Bcl-2 had significantly decreased in HFD mice compared to NOR mice (Fig. 2b). These data indicate that HFD induced endothelial cell apoptosis in the atherosclerosis mouse model.

### Inverse correlation of miR-210 and PDK1 in atherosclerosis ApoE (-/-) mice and in ox-LDL-treated endothelial cells

**Fig. 3:**
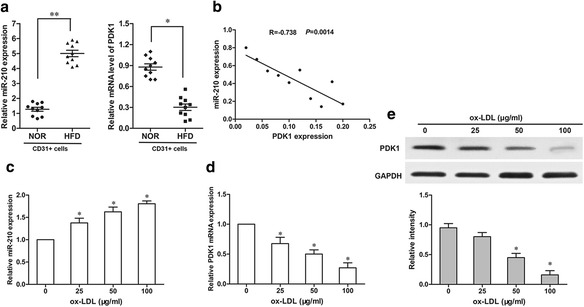
Abnormal expressions of miR-210 and PDK1 were detected in CD31+ endothelial cells and ox-LDL-treated endothelial cells. **a** The expression levels of miR-210 and PDK1 in ApoE (-/-) mice were detected via RT-qPCR. **b** The correlation of miR-210 and PDK1 levels in ApoE (-/-) mice was measured via Peason’s coefficient correlation analysis. The effects of ox-LDL (0, 25, 50 or 100 ug/ml) on miR-210 (**c**) and PDK1 (**d** and **e**) expressions were detected via RT-qPCR and western blot. *p < 0.05, **p < 0.01

miR-210 expression in CD31+ cells from ApoE (-/-) mice was examined using RT-qPCR. As shown in Fig. 3a, a significantly higher expression level of miR-210 was observed in HFD mice than that in NOR mice (*p* < 0.01), while the expression level of PDK1 was remarkably downregulated in HFD mice (*p* < 0.05). Using Pearson’s coefficient correlation analysis, a statistically significant inverse correlation (*R* = −0.738, *P* = 0.0014) was observed between the expressions of miR-210 and PDK1 (Fig. 2b).

Oxidized low-density lipoprotein (ox-LDL), a well-known atherogenic factor, was used to induce endothelial cell apoptosis in this study. HAECs were treated with 0, 25, 50 and 100 ug/ml doses of ox-LDL for 24 h. We found that miR-210 expression was enhanced by different ox-LDL treatments in HAECs in a dose-dependent manner (Fig. 2c). To further investigate the effect of ox-LDL on the expression of PDK1, we measured the mRNA and protein levels of PDK1 in these HAECs. The results showed downregulation of PDK1 in a dose-dependent manner in HAECs compared to the control. These data indicate that miR-210 and PDK1 might be involved in endothelial cell apoptosis in atherosclerosis.

### PDK1 is a target for miR-210 in HAECs

**Fig. 4:**
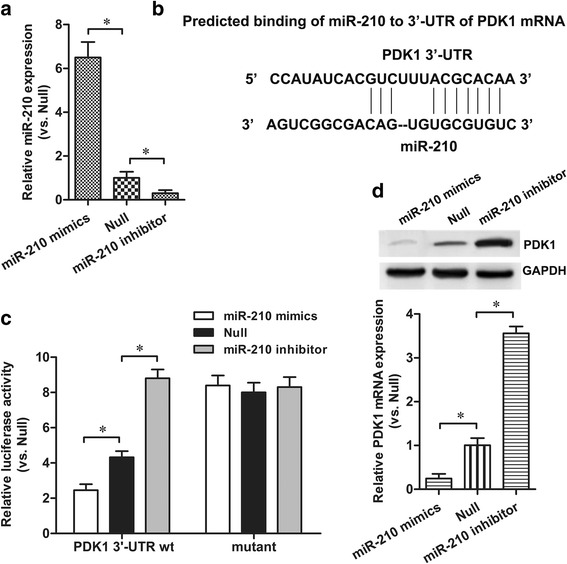
PDK1 was a target for miR-210 in HAECs. **a** miR-210 expression levels in HAECs with miR-210 mimics, miR-210 inhibitor or null control (Null) transfection were detected via RT-qPCR. **b** The binding site of miR-210 to the 3′-UTR of PDK1 was calculated through bioinformatics analysis. **c** Relative luciferase activities with the wild-type or mutant 3′-UTR of PDK1 were quantified. **d** The effect of miR-210 on PDK1 expression was examined via qRT-PCR and western blot. *p < 0.05

To understand the mechanisms underlying miR-210 and PDK1 interaction in atherosclerosis, we further identified the potential target sites of PDK1 to miR-210 (Fig. 4b). Firstly, HAECs were transfected with miR-210 mimics, miR-210 inhibitor or Null for 48 h, and the miR-210 expression alternations were confirmed via RT-qPCR (Fig. 4a). Then, the wild-type or mutant PDK1-3′UTR vectors were co-transfected with miR-210 mimics or miR-210 inhibitor into HAECs. We found that miR-210 mimics markedly inhibited the luciferase activity of the wild-type PDK1-3′UTR reporter (*p* < 0.05), whereas the miR-210 inhibitor increased luciferase activity compared to the negative control (*p* < 0.05) (Fig. 4c). Transfection of miR-210 mimics or miR-210 inhibitor had no significant effect on the luciferase activity of the mutated PDK1-3′UTR reporter.

To confirm the regulatory effect of miR-210 on PDK1 in HAECs, we examined the changes in PDK1 protein level following miRNA transfection. Western blot and RT-qPCR analysis indicated that miR-210 mimics significantly suppressed PDK1 expression both at the transcriptional and translational levels. This was reversed by transfection of miR-210 inhibitor (Fig. 4d). These findings suggest that PDK1 expression is directly regulated by miR-210 in HAECs.

### MiR-210 promotes endothelial apoptosis by targeting PDK1

**Fig. 5:**
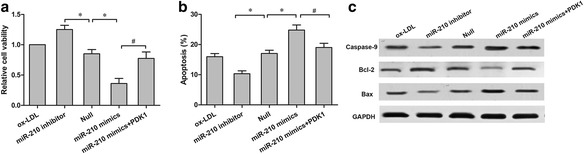
miR-210 promoted endothelial apoptosis by targeting PDK1. **a** HAECs cell viability was determined using the MTT assay. **b** Cell apoptosis of HAECs with different treatments was measured via flow cytometry. **c** The protein levels of Bax, caspase-9 and Bcl-2 in HAECs with different treatments were detected via western blot. *p < 0.05 vs. Null; ^#^ *p* < 0.05 vs. miR-210 mimics

The MTT assay was used to assess the effect of miR-210 on endothelial cell survival. We confirmed that miR-210 mimics significantly decreased the cell viability of ox-LDL-treated HAECs (*p* < 0.05), whereas miR-210 inhibitor significantly increased the viable cell number in ox-LDL-treated HAECs (*p* < 0.05; Fig. 5a). The flow cytometry assay also revealed that are markedly increased proportion of cell apoptosis was observed in the ox-LDL-treated HAECs transfected with miR-210 mimics (*p* < 0.05), whereas transfection with the miR-210 inhibitor was sufficient to abolish ox-LDL-induced apoptosis in ox-LDL-treated HAECs (*p* < 0.05; Fig. 5b).

A rescue experiment was conducted to validate the crucial role of PDK1 in mediating the apoptotic action of miR-210 on HAECs. As shown in Fig. 5a, MTT analysis demonstrated that overexpression of PDK1 significantly abolished the restrained effect of miR-210 mimics on HAEC cell viability (*p* < 0.05, miR-210 + PDK1 vs. miR-210). Flow cytometry further confirmed that PDK1 overexpression increased the survival number of ox-LDL-treated HAECs transfected with miR-210 mimics by reducing cell apoptosis (*p* < 0.05, miR-210 + PDK1 vs. miR-210; Fig. 5b). Similar regulatory changes were observed through western blot detection of pro-apoptosis makers (Fig. 5c). These data suggest that miR-210 promotes ox-LDL-induced apoptosis through down regulation of the expression of PDK1 in HAECs.

### MiR-210 inhibits P13K/Akt/mTOR signaling activation by targeting PDK1 in atherosclerosis

**Fig. 6:**
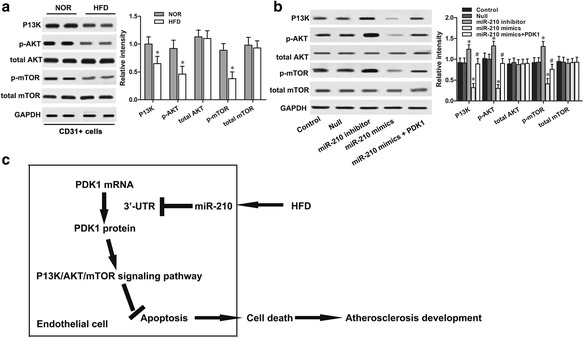
MiR-210 inhibited P13K/Akt/mTOR signaling activation by targeting PDK1 in atherosclerosis. The protein levels of P13K/Akt/mTOR signaling in (**a**) CD31+ endothelial cells of ApoE (-/-) mice and (**b**) ox-LDL-treated HAECs were detected via western blot. **c** A schematic model of miR-210 involved in atherosclerosis-associated endothelial cell apoptosis. *p < 0.05 vs. Null; ^#^ *p* < 0.05 vs. miR-210 mimics

It has been well established that PDK1 activates Akt/mTOR signaling in severe cardiac disease [26]. We were interested in determining the involvement of PDK1 and P13K/Akt/mTOR signaling in the miR-210 regulatory apoptotic effect in atherosclerosis. The expressions of key components in P13K/Akt/mTOR signaling were detected in endothelial cells from ApoE (-/-) mice. Western blot results showed that the expression levels of P13K, p-Akt and p-mTOR significantly decreased in HFD mice compared to NOR mice, while the total Akt and mTOR expression levels in HFD mice did not decrease significantly (Fig. 6a). Furthermore, we transfected ox-LDL-treated HAECs with miR-210 mimic, miR-210 inhibitor, or PDK1-overexpression plasmid. Consistent with the in vivo tissue result, western blot results showed that miR-210 overexpression significantly downregulated P13K, p-Akt and p-mTOR expressions, and the mir-210 inhibitor significantly upregulated P13K, p-Akt and p-mTOR expressions, but had no effect on the total Akt and mTOR protein expressions in ox-LDL treated HAECs (Fig. 6b). The inhibition of P13K/Akt/mTOR signaling by miR-210 mimics could be reactivated by PDK1 transfection. Our results suggest that atherosclerosis-associated endothelial cell apoptosis might result from abundant miR-210 that inhibits P13K/Akt/mTOR signaling activation by directly targeting PDK1.

## Discussion

Atherosclerosis is a complex arterial disease characterized by vascular wall inflammation and atherosclerotic plaque accumulation [1]. Endothelial cells play a critical role in the development of atherosclerosis, so damage to the vascular endothelium might increase the risk of triggering the pathogenesis of atherosclerosis [27]. High glucose and ox-LDL are effective pro-atherosclerotic factors that have been widely applied to induce apoptosis of endothelial cells in both in vivo and in vitro studies [28, 29]. Though therapeutic improvement of atherosclerosis has been achieved over the past decade, the prognostic risk is still high in many dyslipidemia patients, which suggests an urgent for a potent novel therapy [30].

This study demonstrated that aberrant miR-210 expression is involved in the progression of atherosclerosis via stimulation of endothelial apoptosis in vivo and in vitro. Upregulation of miR-210 was associated with increased endothelial cell apoptosis and an inverse correlation was found between miR-210 and PDK1 mRNA expressions. The dual-luciferase activity assay further verified that the 3′-UTR of PDK1 was a directly binding target to miR-210. Overexpression of PDK1 was sufficient to reverse miR-210-induced apoptosis in ox-LDL-treated HAECs. We also revealed the underlying mechanisms involved in the pathology of atherosclerosis: the downstream apoptotic pathway P13K/Akt/mTOR activation was inhibited by miR-210, as schematically summarized in Fig. 6c. This study provides convincing evidence that miR-210 might be a potent therapeutic target for atherosclerosis.

Recent reports have emphasized that abnormally expressed miRNAs participate in the mediation of endothelial cell destiny [31]. In fact, miR-210 has been previously reported to be involved in various cardiovascular diseases. Chen et al. suggested that increased miR-210 expression was found in the aorta of mice with high-fat diets and that it increased the incidence risks of cardiovascular disease and gastrointestinal cancer [32]. Zaccagnini et al. reported that miR-210 was a crucial element in the adaptive mechanism to regulate oxidative metabolism and oxidative stress in the acute peripheral ischemia [33]. Zhao et al. proved that serum miR-210 expression was close to the fetal level in Chinese adult patients with chronic heart failure [34]. Lou et al. indicated that miR-210 overexpression was associated with the regulation of angiogenesis in response to ischemic injury to the brain [35]. Li et al. and Raitoharju et al. also demonstrated that miR-210 was a potential biomarker for diagnosis of atherosclerosis [24, 25]. Our study further identified that the regulatory mechanisms of miR-210 in atherosclerosis pertain to the promotion of endothelial apoptosis.

Previous studies have declared that the mechanisms underlying miRNA regulation of endothelial apoptosis are often attributed to targeting critical genes or the pivotal pathways related to apoptosis [36, 37]. Here, we discovered that miR-210 regulated endothelial apoptosis by targeting PDK1. 3-phosphoinositide-dependent protein kinase-1 (PDK1) is a 556-amino acid kinase that possesses a C-terminal pleckstrin homology domain [38]. Its character as a potential anticancer target relates to the phosphorylation of a series of protein kinases, including apoptosis regulatory factor protein kinase B (PKB/Akt) and protein kinase C (PKC), which play crucial roles in the regulation of physiological processes of cell survival and death [39]. In our study, we found that reversing PDK1 expression could significantly resist miR-210-induced cell apoptosis in ox-LDL-treated HAECs, implying an anti-apoptotic role of PDK1 in the development of atherosclerosis.

Functional analysis also revealed that the activation of the P13K/Akt/mTOR signaling pathway was notably suppressed by miR-210 in apoptotic endothelial cells, but was reactivated by exotic PDK1 transfection. The aberrant activation of the P13K/Akt/mTOR pathway was associated with numerous human disorders [40]. Accumulating data from biological studies demonstrate that the P13K/Akt/mTOR pathway plays a prominent role in cell survival, metabolism, growth and proliferation by directly regulating apoptotic proteins such as Bcl-2, caspase-3, caspase-9 and Bax [41, 42].

In ApoE (-/-) mice with HFD-induced atherosclerosis, the elevated expression of pro-apoptosis factors caspase-3, caspase-9 and Bax and the decreased level of anti-apoptosis factor Bcl-2 were induced by miR-210 overexpression. Effect of caspases and Bcl-2/Bax involvement contributes to the initiation of the mitochondrial pathway of apoptosis [43, 44]. These findings might account for the severe cell death levels in atherosclerosis.

## Conclusions

Our study identified the pro-atherosclerotic role of miR-210 by promoting endothelial apoptosis partially through targeting PDK1 to suppress the P13K/Akt/mTOR signaling pathway, providing new insight into the treatment of atherosclerosis.

## Abbreviations

ApoE: Apolipoprotein E
cDNA: Complementary DNA
DMSO: Dimethyl sulfoxide
HAECs: Human aortic endothelial cells
HFD: High-fat diet
HRP: Horseradish peroxidase
miRNA: microrna
mTOR: Mammalian target of rapamycin
MTT: 3-(4,5-cimethylthiazol-2-yl)-2,5-diphenyl tetrazolium bromide
ox-LDL: Oxidized low-density lipoprotein
P13K/Akt: Phosphatidylinositol 3-kinase/protein kinase B
PDK1: 3-phosphoinositide-dependent protein kinase-1
PVDF: Polyvinylidene fluoride
SDS-PAGE: Sodium dodecyl sulfate-polyacrylamide gel electrophoresis
UTR: Untranslated region
